# Rare circle of Willis variants associated with multiple intracranial aneurysms and subarachnoid hemorrhage: A case report

**DOI:** 10.1097/MD.0000000000050402

**Published:** 2026-08-28

**Authors:** Mohamad H. Mosi, Hisham Alfarra, Nawwar Soliman, Moaaz Khalouf Alshaar, Mahmoud Khalouf Alshaar, Ghassan Hamzeh

**Affiliations:** a Department of Internal Medicine, National University Hospital and Al Mouwasat University Hospitals, Damascus University, Faculty of Medicine, Damascus, Syria; b Department of Radiology, Hama National Hospital, Hama, Syria; c Department of Internal Medicine, National University Hospital and Al Mouwasat University Hospitals, Damascus University, Faculty of Medicine, Damascus, Syria; d Faculty of Medicine, Hama University, Hama, Syria; e Department of Pathology, Hama National Hospital, Hama, Syria; f Department of Neurology, National University Hospital, Damascus University, Faculty of Medicine, Damascus, Syria.

**Keywords:** Circle of Willis, posterior cerebral artery, posterior communicating artery, ruptured aneurysm, subarachnoid hemorrhage

## Abstract

**Rationale:**

Subarachnoid hemorrhage from a ruptured intracranial aneurysm is a type of stroke with high rates of mortality and disability, and the prevalence of aneurysm formation increases in the presence of Circle of Willis variants.

**Patient concerns:**

A 64-year-old man with a history of hypertension and psoriatic arthritis presented with a sudden severe headache and walking difficulties without any loss of consciousness.

**Diagnoses:**

Non-contrast computed tomography showed a diffuse subarachnoid hemorrhage. Computed tomography angiography demonstrated multiple intracranial aneurysms together with variants of the posterior communicating artery and posterior cerebral artery, which may have contributed to aneurysm formation.

**Interventions:**

The patient was started on oral Nimodipine to prevent cerebral vasospasm and underwent stent-assisted coil embolization of the ruptured aneurysm.

**Outcomes:**

His symptoms and clinical condition improved a few days after the embolization, and he was discharged after 18 days.

**Lessons:**

This is the first documented case report from Syria, and possibly the first ever, detailing such Circle of Willis variants associated with cerebral aneurysms. It underscores the need for further studies exploring the relationship between the type of variants and the number and size of cerebral aneurysms, which is important for a better understanding of the epidemiology of cerebral aneurysms.

## 1. Introduction

The Circle of Willis (COW) is an anastomotic network of arteries at the base of the brain that provides collateral blood perfusion to prevent ischemia.^[1]^ Vessels forming the COW are the following: the bilateral anterior cerebral arteries, the bilateral internal carotid arteries, the anterior communicating artery, the bilateral posterior cerebral arteries, the bilateral posterior communicating arteries, and the basilar artery.^[2]^

The estimated prevalence of variations of unilateral posterior communicating artery (PComA) in healthy people is about 68 ± 14%.^[3]^ Some variations are associated with some pathologic conditions such as migraine,^[4]^ ischemic stroke^[5]^ and the formation and rupture of intracranial aneurysms.^[3]^

The intracranial aneurysms are acquired dilations of the brain vessels that occur in 1–2% of the population. The most dangerous complication of an intracranial aneurysm is rupture, which accounts for 80–85% of nontraumatic subarachnoid hemorrhage with a high mortality rate.^[6]^ Many risk factors have been identified with the rupture of intracranial aneurysms, including age, size, and location.^[7]^ The most common locations for intracranial aneurysms and their rupture are the anterior communicating artery (AComA) and the posterior communicating artery (PComA).^[8,9]^ Aneurysmal rupture leads to intracranial hemorrhage, with SAH carrying particularly high rates of mortality and disability.^[10]^

In this case report, we discuss a subarachnoid hemorrhage in a male patient caused by the rupture of an intracranial aneurysm associated with variations of the Circle of Willis.

## 2. Case Report

**Figure 1.**
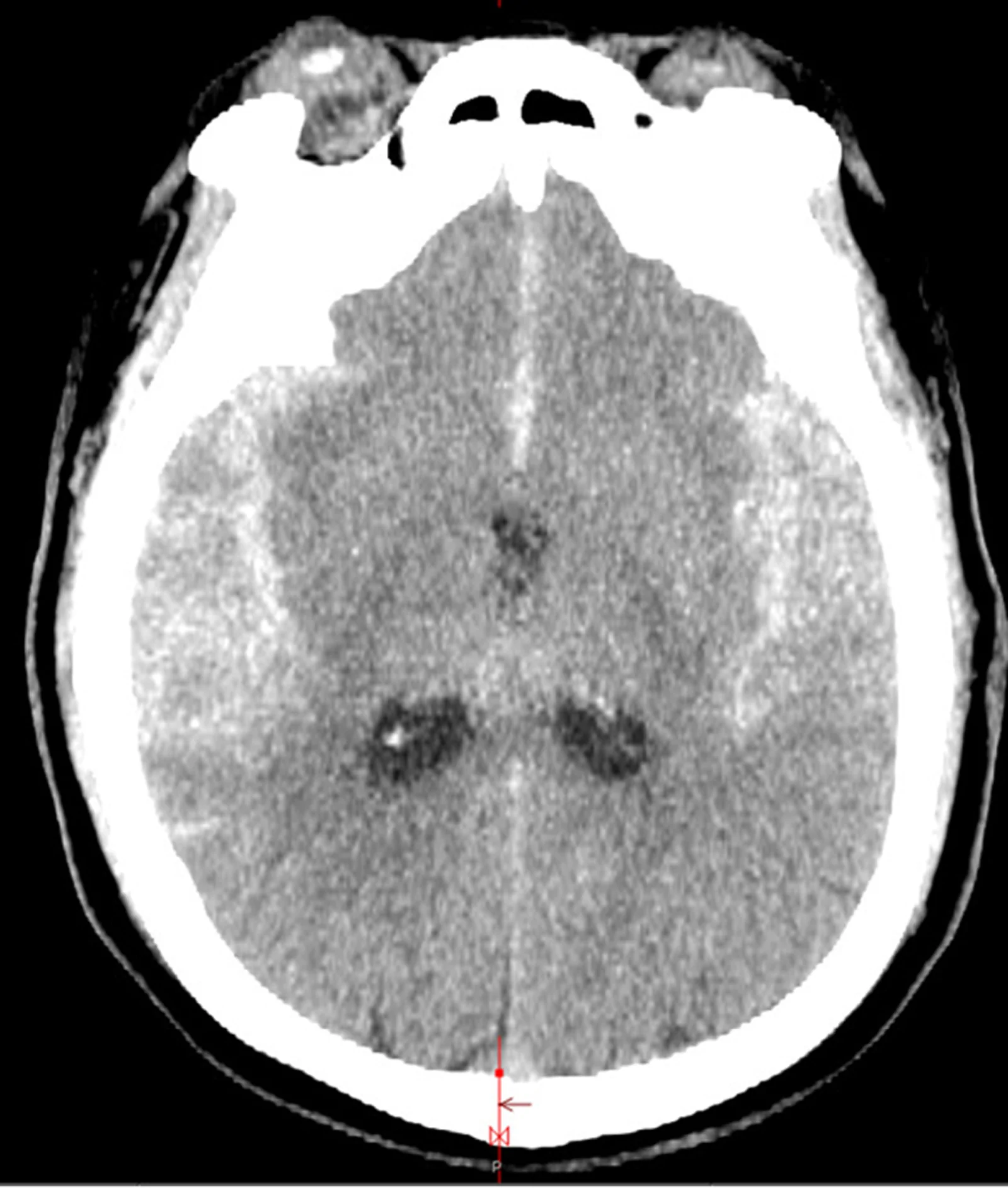
A CT scan of the head without contrast (axial) shows diffuse subarachnoid hemorrhage.

A 64-year-old man was admitted to the ER department at another hospital due to severe headache, nausea, vomiting, and dizziness. The symptoms started suddenly at 3 am, after sleeping normally. The patient was awake and suffered from emesis, neck pain, photophobia, and speaking and walking difficulties that required assistance to go to the hospital, with no seizures or loss of consciousness. The patient appeared ill and uncomfortable at the ER; his blood pressure was 140/73 mm Hg. The physical examination was unremarkable, except for neck stiffness. A brain computed tomography (CT) scan showed diffuse subarachnoid hemorrhage (Fig. 1).

The patient was transferred to our hospital for intensive care admission after performing a head and neck CT angiography (CTA). The patient has a history of hypertension, psoriasis with psoriatic arthritis, and gout, and uses allopurinol, lisinopril, and dexamethasone. The family history was unremarkable. On admission to our hospital, the patient was afebrile and uncomfortable, and his blood pressure was 140/76 mm Hg, heart rate was 67 beats/minute and regular, respiratory rate was 16/minute, and his saturation was 98% in room air. The physical examination was unremarkable. The neurological examination demonstrated a Glasgow Coma Scale (GCS) score of 15/15, equal and reactive pupils, normal reflexes in all four limbs, and nuchal rigidity without focal neurological deficits.

**Figure 2.**
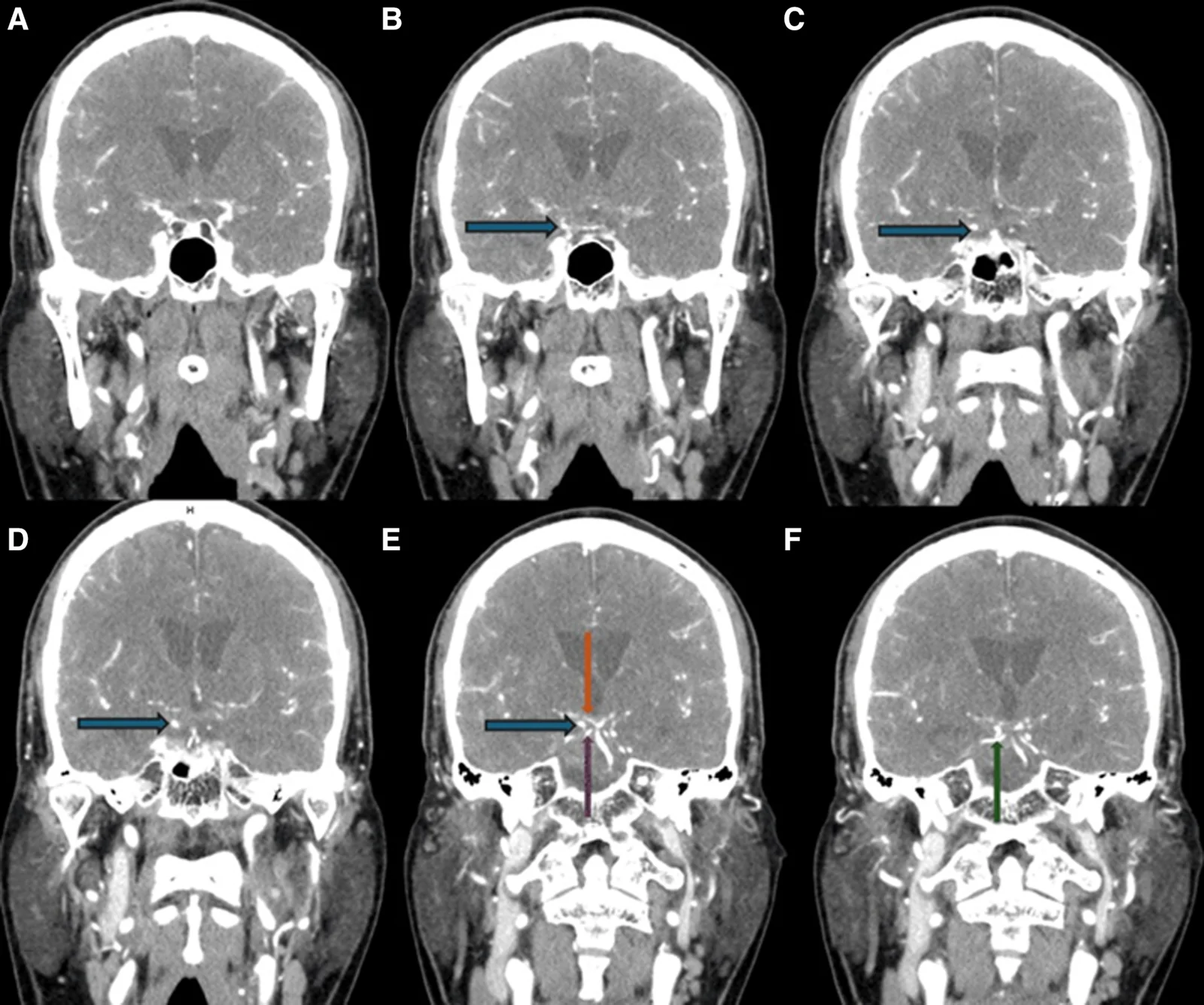
CTA coronal view slides reveal the course of the right posterior communicating artery (blue arrow) from the right internal carotid artery to its combination with the right superior cerebellar artery (purple arrow). Right posterior cerebral artery P1 (orange arrow). The right posterior communicating artery combines with the right superior cerebellar artery (green arrow).

The initial laboratory tests are shown in Table 1. An electrocardiogram was performed and showed no abnormalities. On admission, the CTA of the head and neck demonstrated the responsible vascular lesions. The CTA of the head showed a 3.6 × 4.6 mm aneurysm of the superior cerebellar artery (SCA) at the point where the posterior communicating artery (PComA) connects to the SCA, before the P2 segment of the posterior cerebral artery (PCA) emerges from the SCA; a 2 × 1.5 mm aneurysm of the P2 segment of the left PCA; and a 2 mm aneurysm of the left PComA (Fig. 2). The right PComA connected the SCA and the internal carotid artery (ICA), the right P2 of the PCA originated from the right SCA, and the right P1 was occluded at its origin. The CTA of the neck showed 25% stenosis of the right carotid artery, with the other arteries of normal appearance. The patient was started on intravenous fluids and oral Nimodipine (60 mg every 4 hours for 21 days).

**Table 1:** The initial laboratory tests at admission (12/09/2019).

WBC	14	Na	135	TP	6.6
L/N	18/75	**K**	4.2	**Alb**	3.9
Hb	16	**Cl**	102	**TB**	0.9
Hct	49	**Glu**	161	**ALP**	101
Plt	244	**AST**	28	**INR**	1
Cr	0.9	**ALT**	25	**PT**	100%
Ur	33	**Ca**	9.3	**BMI**	30
CSF results (12/09/2019)					
**Appearance**	**RBC**	**WBC**	**L/N**	**Glu**	**Protein**
Cloudy Red	80,500	171	44/42	55	82

AST: Aspartate transaminase, normal range (8–33 U/L).

ALT: Alanine transaminase, normal range (4–36 U/L).

WBC: white blood cells; RBC: red blood cells; Hb: hemoglobin; Hct: hematocrit; N: neutrophils; L: lymphocytes; Plt: platelets; Glu: glucose; Ur: urea; Cr: creatinine; Na: sodium; K: potassium; Cl: chloride; TP: total proteins; Alb: albumin; Ca: calcium; PT: prothrombin time; INR: international normalized ratio; TB: total bilirubin; ALP: alkaline phosphatase; BMI: body mass index; CSF: cerebrospinal fluid.

Shortly after admission, the patient underwent stent-assisted coil embolization of the ruptured aneurysm and was admitted to the ICU for close monitoring of his vital signs and laboratory values. He had been kept NPO (nothing by mouth) for 8 hours before the procedure. On the second day after embolization, he developed atrial fibrillation (heart rate 110 beats/minute, irregular) and was started on metoprolol for rate control, which was achieved without anticoagulation.

**Figure 3.**
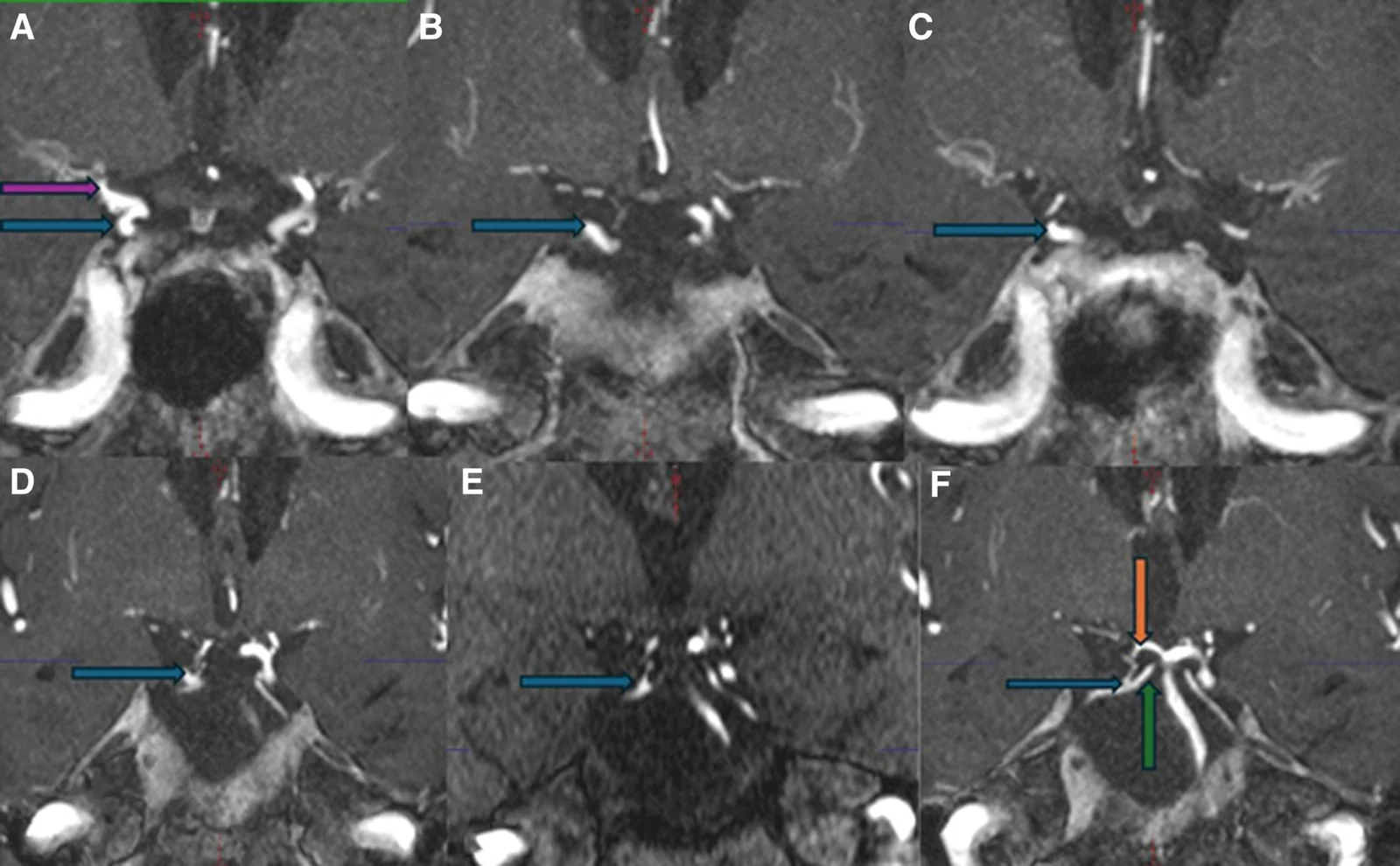
MRA image: MRA coronal view slides (A to F) show the course of the right posterior communicating artery (blue arrow) from its origin, which is the right internal carotid artery (purple arrow). Slides from D to F reveal the right posterior communicating artery (blue arrow) combining with the right superior cerebellar artery (green arrow). The right posterior cerebral artery P1 (orange arrow) is stenotic at its end (slide F).

**Figure 4.**
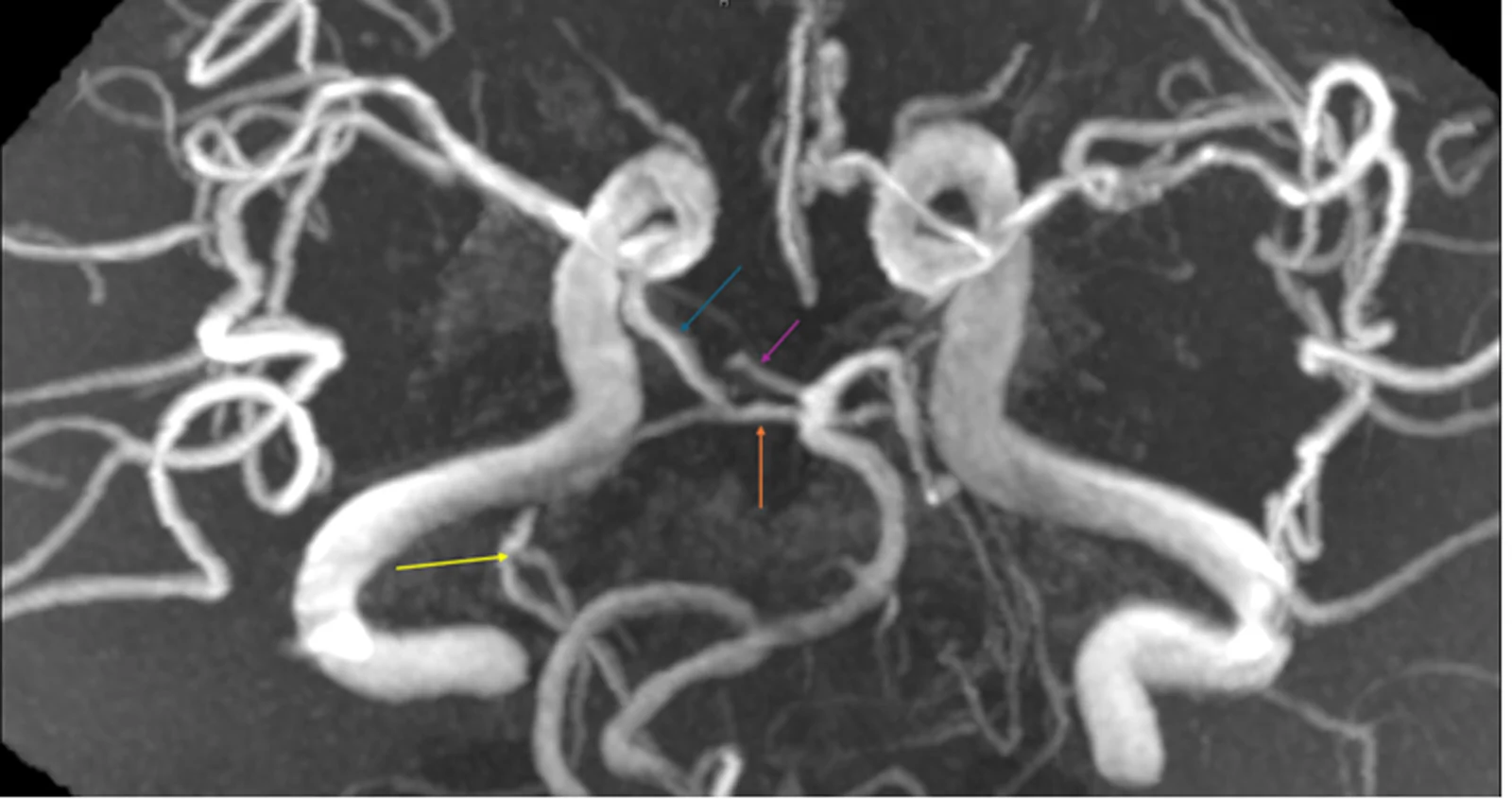
MRA (3D Reconstruction). Blue arrow: Right posterior communicating artery, Purple arrow: Right posterior cerebral artery (P1), Orange arrow: Right superior cerebellar artery, Yellow arrow: The point where the posterior cerebral artery (P2) originates from the right superior cerebellar artery.

**Figure 5.**
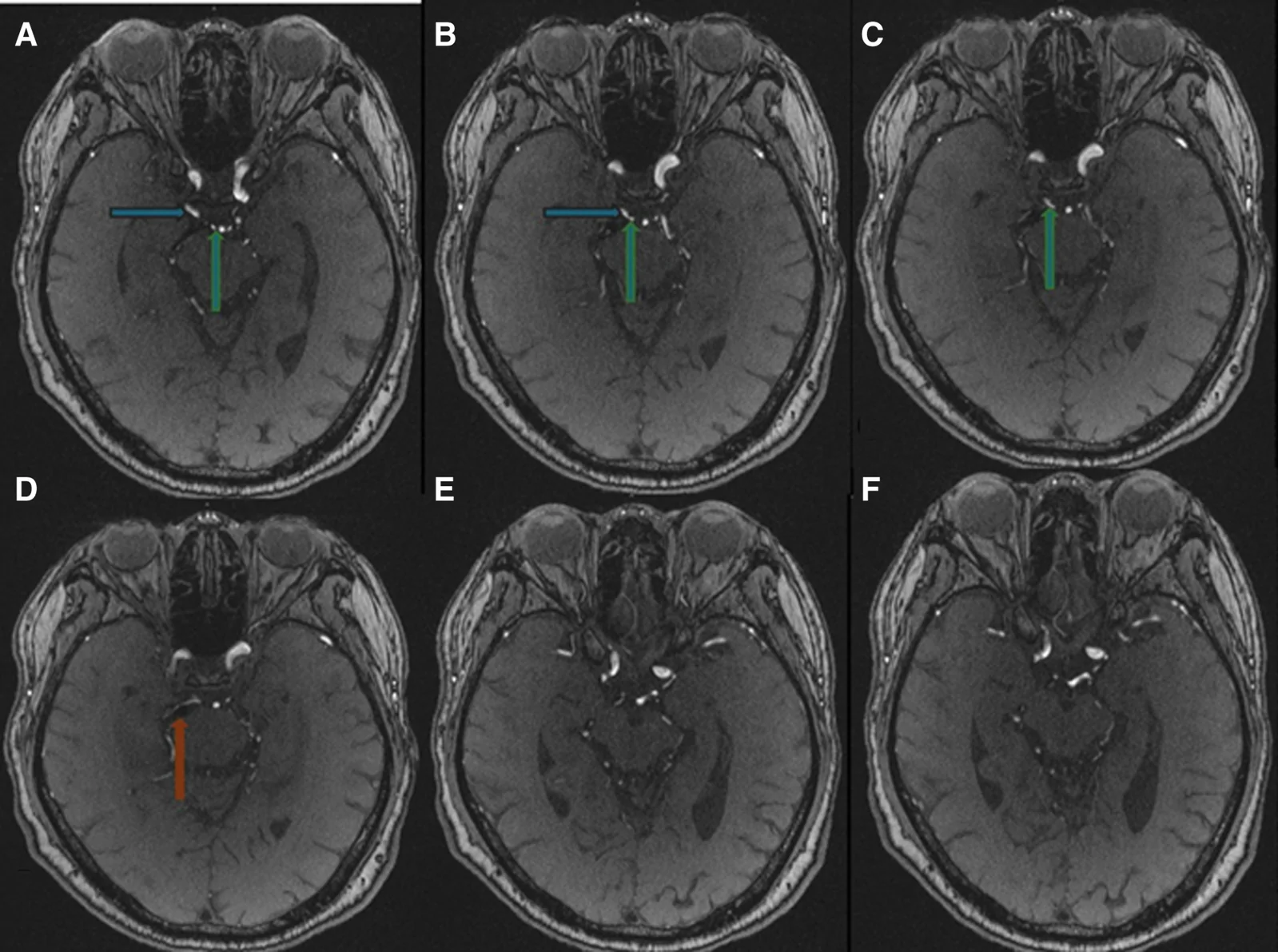
MRA axial view slides after aneurysm coiling (red arrow). Slide A shows the right posterior communicating artery (blue arrow) and the right superior cerebellar artery (green arrow). Slide C reveals the combining point (green arrow) while the basilar artery is still present before its bifurcation. The orange arrow in slide D points to the coiling area.

After embolization, magnetic resonance angiography (MRA) was performed and confirmed the variant anatomy: the right PComA connected the right SCA and the right ICA, the right P2 of the PCA arose from the SCA, and the right P1 was occluded (Figures 3 and 4). The patient’s symptoms and neurological examination improved, and he was discharged after 18 days. A follow-up MRA obtained 3 months after coiling is shown in Figure 5.

## 3. Discussion

The Circle of Willis (COW) is an anastomotic ring of arteries that lies in the interpeduncular fossa at the base of the brain and provides constant, regular cerebral blood flow to prevent ischemia. The vascular ring is composed of bilateral anterior cerebral arteries (ACAs), the anterior communicating artery (AComA), and bilateral internal carotid arteries (ICAs), which together form the anterior circulation (carotid system). The posterior circulation (Vertebrobasilar System) is composed of bilateral posterior communicating arteries (PComAs), bilateral posterior cerebral arteries (PCAs), and the basilar artery.^[1]^

This typical anatomical configuration of COW may have some variations, and these variations have been studied for their connections with the development and severity of cerebrovascular diseases.^[11]^ Vessels generally vary in caliber; often, they are hypoplastic, duplicated, or even absent.^[12]^ Four criteria are classically used to determine normal (non-variant) anatomy of the COW^[13]^: all segments are present, all segments arise from their natural origins, no accessory arteries are present, and all segments have an external diameter of > 1 mm.

Assessed against the four criteria for a normal (non-variant) Circle of Willis, our patient’s right posterior circulation was abnormal on 2 counts: not all segments were present – the right P1 segment of the PCA was occluded at its origin – and the remaining segments did not arise from their natural origins, as the right P2 arose from the SCA and the right PComA connected the ICA to the SCA rather than to the PCA. With the right P1 occluded, the right posterior cerebral territory was supplied not from the basilar artery but from the carotid circulation through an enlarged PComA and the SCA – a configuration at the extreme end of the fetal-type spectrum.

Intracranial aneurysms (IAs) are common acquired lesions, occurring in 1–2% of the population.^[6]^ Size and location are established predictors of formation and rupture,^[14]^ but local hemodynamics – particularly wall shear stress (WSS) – are increasingly recognized as central to both aneurysm initiation and rupture,^[15–17]^ which is especially relevant here given the patient’s markedly altered Circle of Willis.

The COW has a complex hemodynamic system with a constant blood flow to prevent ischemia. The blood flow velocity and the intraluminal pressure in the proximal or distal parts of COW are influenced by the systemic blood pressure and the configuration of COW.^[18–20]^ Also, the flow rate in the COW can be influenced by either the efferent resistance distribution or the radius of all the segments in COW, and the variations of COW have been associated with multi-hemodynamic changes which can raise the prevalence of aneurysm formation and rupture.^[21]^

Circle of Willis variants redistribute flow in ways that favor aneurysm formation: a meta-analysis found that fetal-type or enlarged PComA configurations are associated with increased aneurysm formation and rupture, attributed to elevated WSS at reconfigured junctions.^[22]^ In our patient, the ruptured aneurysm that caused the SAH – a 3.6 × 4.6 mm aneurysm of the SCA at the PComA–SCA junction – lay at the site of this anomalous confluence. With the right P1 occluded, the entire right posterior cerebral territory was perfused through the PComA and SCA, converting the normally low-flow PComA into a primary conduit. Such fetal-type configurations generate a high-flow, high-shear inflow jet at the PComA bifurcation,^[23]^ and high WSS with a positive gradient at a branch point is the condition implicated in aneurysm initiation.^[16]^ We therefore propose that redirection of posterior cerebral inflow through the PComA–SCA junction concentrated WSS at that site, predisposing to the culprit aneurysm whose rupture produced the SAH. Given the single-case design, this mechanism remains hypothesis-generating and would be best confirmed with patient-specific flow modeling.

SAH is a life-threatening emergency with high rates of mortality and disability, and roughly 30% of patients die within the first days to weeks after the initial hemorrhage^[10]^; intracranial aneurysms and SAH together account for about 5% of all strokes.^[24,25]^ Its complications are numerous, the most important being cerebral vasospasm, which occurs in about 70% of patients.^[26]^ Vasospasm begins after the third day of hemorrhage, peaks at days 7–8, and can cause delayed cerebral ischemia (DCI), which adversely affects survival.^[26,27]^ Cardiac arrhythmias are also common, most often QT prolongation and deep T-wave inversions, with atrial fibrillation and torsades de pointes being less frequent.^[28,29]^ In our patient, atrial fibrillation developed and was managed with rate control alone (metoprolol 50 mg every 8 hours for 21 days), without anticoagulation.

Our patient, after being diagnosed with subarachnoid hemorrhage, was admitted to the ICU with close monitoring of his vital signs and was prepared for embolization. During his admission, the patient received oral Nimodipine 60 mg every 4 hours for 21 days to prevent cerebral vasospasm because Nimodipine is the only pharmacologic treatment currently used to prevent vasospasm.^[30]^

Endovascular coiling is considered the preferred treatment for ruptured cerebral aneurysms, with a significant reduction in 1-year mortality compared with neurosurgical clipping, although it carries a higher risk of rebleeding.^[31]^ Our patient underwent endovascular embolization and was transferred to the ICU, with improvement of his symptoms and neurologic examination; he was discharged after 18 days.

## 4. Conclusions

This is the first documented case report from Syria, and possibly the first ever, detailing this type of COW variant associated with cerebral aneurysms. This case underscores the need for further studies to explore the relationship between the type of variants and the number and size of cerebral aneurysms. Such research is crucial for a better understanding of the epidemiology of cerebral aneurysms.

## Acknowledgments

The authors thank the patient for consenting to the publication of this case report. This case is reported in accordance with the CARE guidelines.

## Author contributions

**Writing – original draft:** Mohamad H. Mosi, Hisham Alfarra, Nawwar Soliman, Moaaz Khalouf Alshaar.

**Writing – review & editing:** Mohamad H. Mosi, Hisham Alfarra, Nawwar Soliman, Moaaz Khalouf Alshaar, Mahmoud Khalouf Alshaar.

**Supervision:** Ghassan Hamzeh.

## Correction

In the published version, Figures 2, 3, and 4 have been placed in the wrong positions. Consequently, we have swapped these figures into their correct positions: Figure 4 is now Figure 2, Figure 2 is now Figure 3, and Figure 3 is now Figure 4.
